# A Localization and Tracking Approach in NLOS Environment Based on Distance and Angle Probability Model

**DOI:** 10.3390/s19204438

**Published:** 2019-10-14

**Authors:** Xin Tian, Guoliang Wei, Jianhua Wang, Dianchen Zhang

**Affiliations:** 1 Department of Control Science and Engineering, University of Shanghai for Science and Technology, Shanghai 200093, China; 171560040@st.usst.edu.cn (X.T.); 171560042@st.usst.edu.cn (J.W.); 172560460@st.usst.edu.cn (D.Z.); 2 College of Science, University of Shanghai for Science and Technology, Shanghai 200093, China

**Keywords:** NLOS, EKF, localization, probability model, MLE

## Abstract

In this paper, an optimization algorithm is presented based on a distance and angle probability model for indoor non-line-of-sight (NLOS) environments. By utilizing the sampling information, a distance and angle probability model is proposed so as to identify the NLOS propagation. Based on the established model, the maximum likelihood estimation (MLE) method is employed to reduce the error of distance in the NLOS propagation. In order to reduce the computational complexity, a modified Monte Carlo method is applied to search the optimal position of the target. Moreover, the extended Kalman filtering (EKF) algorithm is introduced to achieve localization. The simulation and experimental results show the effectiveness of the proposed algorithm in the improvement of localization accuracy.

## 1. Introduction

With the rapid development of wireless communication technology in recent years, wireless localization systems have received considerable research interest due to the increasing demand of location-based services (e.g., [1,2,3,4,5]). At present, many localization technologies have been widely used, such as GPS [6], CPS [7], and WLAN [8,9]. It is acknowledged that these localization systems perform well under ideal conditions [10,11,12,13]. However, in real-world situations, due to the complexity of the environment such as the non-line-of-sight (NLOS) condition, the obstruction of the line of sight of the wireless communication between the anchor and the target may decrease the accuracy of the distance measurement, which accordingly leads to a poor localization accuracy [14].

So far, there are various methods to deal with ranging bias in the NLOS propagation. Based on the range measurements, the target localization issue in harsh indoor environments was investigated in [15]. By using the semi-definite programming relaxation technique, a robust estimator was introduced in [16] for the purpose of coping with the NLOS bias. The localization accuracy of range-only sensors with both additive and multiplicative noises was investigated in [17,18]. After that, in [19], the Manhattan distance was introduced to the WKNN algorithm to distinguish the influence of different reference nodes. In [20], the scene analysis approach was employed including two stages, namely, the offline stage and the online stage. In the offline stage, the wireless signals from all the anchors are recorded at each specific location in order to build a fingerprint database. In the online stage, according to the built fingerprint database, the K-nearest neighbor and weighted K-nearest neighbor algorithms were adopted to classify the data obtained from the environment into specific classifications. On the other hand, most of the existing results with respect to various NLOS identification methods (i.e., distinguish that the environment is in LOS (line-of-sight) or NLOS propagation) are based on range estimates [21,22,23,24,25,26,27]. Note that all these methods require a sufficient number of measurements to reduce the impact of NLOS range estimates, which imposes a great burden on the computational complexity.

Generally, LOS propagation is more likely to occur when the distance between a target and an anchor is short enough. However, in real environments, with the increasing distance between the target and the anchor, the probability of NLOS propagation increases accordingly. In [28], LOS/NLOS probability was assumed to be fixed and known a priori, which ignores the fact that the occurrence probability of LOS and NLOS propagations is dependent on the distance between the target and the anchor. In [14], the distance-related LOS/NLOS probability model was proposed. However, in addition to the distance, the NLOS propagation probability is also affected by the angle between the target and the anchor, which has not been taken into consideration in most of the reported results.

In this paper, the localization problem is considered in a real environment. The main contributions of this paper are summarized as follows:

(1) An optimization algorithm based on a distance and angle probability model for an indoor NLOS environment is proposed. Based on the NLOS propagation occurrence probability model, the maximum likelihood estimation (MLE) method is used to estimate the target position in order to suppress the NLOS error. In order to increase the speed of the operation, a simplified Monte Carlo algorithm is used to estimate the state. In addition, the extended Kalman filtering (EKF) algorithm is applied to the localization system to reduce measurement errors.

(2) The parameter acquisition method of the NLOS propagation occurrence probability model is given, which is obtained by sampling in the environment to acquire the probability of occurrence of NLOS status at different distances and angles.

(3) According to the characteristics of the signal, a simulation environment is established, which is similar to the real environment. A practical measurement scheme is used to verify the effectiveness of the proposed algorithm and compared with the existing algorithms.

The remainder of this paper is organized as follows. In Section 2, the system model is introduced. A probability model of the occurrence of NLOS propagation is formulated in Section 3. Based on the established model in Section 3, an MLE method is presented in Section 4. In Section 5, a localization method based on the extended Kalman filter algorithm is put forward. Simulation and experimental results are given in Section 6. Conclusions are drawn in Section 7.

## 2. System Model

In this paper, the system model under consideration is in a two-dimensional environment. It is assumed that there are *N* anchors in the environment, and the position of the *i*-th anchor is denoted as $\left(x_{i},y_{i}\right)$, where $x_{i}$ is the value of the anchor on the *x*-axis and $y_{i}$ is the value on the *y*-axis. Similarly, the location of the target is represented as (x,y). The measurement distance between the *i*-th anchor and the target is expressed as:

(1) $$z_{i}\left(t\right)=d_{ri}\left(t\right)+Gau\left(t\right)+\beta_{i}\cdot NLOS\left(t\right),$$

where $z_{i}\left(t\right)$ is the measured distance and $d_{ri}\left(t\right)$ is the real distance between the *i*-th anchor and the target at time *t*. Gau(t) is the Gaussian white noise with zero-mean and variance $\sigma_{los}^{2}$ in the LOS propagation. NLOS(t) stands for the error in NLOS propagation, which can also be described by the Gaussian white noise with mean $\mu_{nlos}$ and variance $\sigma_{nlos}^{2}$. Here, the Gau(t) and NLOS(t) are assumed to be mutually independent in the propagation environment.

**Remark** **1.** *Because of the complicated propagation environment, not all the propagations are LOS propagations. In (1), $\beta_{i}$ is a random variable taking values with 0 and 1. If the distance is measured in the NLOS propagation, the noise includes Gau(t) and NLOS(t) (i.e., $\beta_{i}=1$). Otherwise, if the distance is measured in the LOS propagation, the noise only includes Gau(t) (i.e., $\beta_{i}=0$).*

It should be pointed out that in practical engineering, the distributions of Gau(t) and NLOS(t) can be determined a priori via the measurements and pre-calibration. Consequently, the probability density function of the $d_{i}\left(t\right)$ in the LOS and NLOS propagation are, respectively, expressed as

(2) $$\begin{array}{cc} p_{los}\left(z_{i}\right) & =\frac{1}{\sqrt{2\pi \sigma_{los}^{2}}}\cdot e^{-\frac{{\left(z_{i}-d_{ri}\right)}^{2}}{2\sigma_{los}^{2}}} \end{array}$$

(3) $$\begin{array}{cc} p_{nlos}\left(z_{i}\right) & =\frac{1}{\sqrt{2\pi (\sigma_{los}^{2}+\sigma_{nlos}^{2})}}\cdot e^{-\frac{{\left(z_{i}-d_{ri}-\mu_{nlos}\right)}^{2}}{2(\sigma_{los}^{2}+\sigma_{nlos}^{2})}}. \end{array}$$

Moreover, it is assumed that NLOS propagation occurs with probability $p_{i,nlos}$. As such, the probability density function of $z_{i}\left(t\right)$ can be expressed as:

(4) $$\begin{array}{cc} p(z_{i};x) & =p_{nlos}\left(z_{i};x\right)\cdot p_{i,nlos}\left(z_{i},\theta_{i};x\right)\\ \; & \;\;\;+p_{los}\left(z_{i};x\right)\cdot p_{i,los}\left(z_{i},\theta_{i};x\right), \end{array}$$

where $\theta_{i}\in \left(-180,180\right]$ represents the angle between the positive direction of the *x*-axis and the vector constituted by the *i*-th anchor and point *x*, and $p_{i,los}\left(z_{i},\theta_{i};x\right)=1-p_{i,nlos}\left(z_{i},\theta_{i};x\right)$.

## 3. NLOS Propagation Occurrence Probability Model

Due to the complexity and uncertainty of the propagation, it is improper to describe the probability of the NLOS propagation as a constant. In order to better reflect reality, it is necessary to extract samples to get the characteristics of the environment. The distance-based probability model was proposed in [14], which described that the probability of the NLOS propagation increased with the distance. However, in the actual environment, the probability of NLOS propagation is related to not only the distance but also the angle. In addition, the probability of NLOS propagation is diverse in different environments. Therefore, a probability model of NLOS propagation needs to be established which gives the probability of NLOS propagation based on the distance and angle information between the measured target and the anchor node. For convenience, it is assumed that the distance and the angle are mutually independent in the NLOS propagation.

Denote by *N* the number of sampling points. In order to reduce the influence of the noise of the LOS propagation in the measurement, multiple distances are measured and the average value is calculated at each sampling point. The actual distance and the measured distance between the *i*-th anchor and the *j*-th sampling point are expressed as $d_{r,i,j}$ and $z_{i,j}$, respectively. $\theta_{r,i,j}\in \left(-180,180\right]$ is the angle between the positive direction of the *x*-axis and the vector which is the anchor to the sampling point. By calculating the difference between the actual distance and the measured distance, we can determine whether NLOS propagation exists or not. As such, an indicative variable is introduced as follows:

(5) $$\begin{array}{cc} g_{i,j} & =\left\{\begin{array}{c} 1,\;\;\;z_{i,j}-d_{r,i,j}>d_{threshold}\\ 0,\;\;\;else \end{array}\right., \end{array}$$

where $g_{i,j}=1$ means that the measured distance is in the NLOS propagation between the *i*-th anchor and the *j*-th sampling point, and $d_{threshold}$ is a given constant, which can be set slightly larger than measurement error. The bound of the measurement error η can be given, and we have

(6) $$\begin{array}{c} -\eta \leq Gau(t)\leq \eta . \end{array}$$

The probability satisfying (6) can be expressed as

(7) $$\begin{array}{c} \zeta =\int_{-\eta }^{\eta }\frac{1}{\sqrt{2\pi }\sigma_{los}}\exp\left(-\frac{z^{2}}{2\sigma_{los}^{2}}\right)dz. \end{array}$$

In order to reduce the possibility that the measurement noise is determined as the NLOS error, the parameter ζ can be set to 0.99, that is, the probability that the measurement error is determined as the NLOS error is 1%. According to (7) and the properties of the Gaussian noise, we can obtain $\eta \approx 2.58\sigma_{los}$. Therefore, $d_{threshold}$ can be set to $2.58\sigma_{los}$.

The maximum of the actual distance between the *i*-th anchor and each sampling point is expressed as $d_{r\max,i}$. Divide the interval $\left(0,d_{r\max,i}\right]$ into $n_{di}$ equal subintervals and the $i_{ds}$-th subinterval is $\left(\left(i_{ds}-1\right)\cdot \frac{d_{r\max,i}}{n_{di}},i_{ds}\cdot \frac{d_{r\max,i}}{n_{di}}\right]$, where $i_{ds}=1,2,\cdots ,n_{di}$. The central point of the $i_{ds}$-th distance subinterval can be expressed as

(8) $$\begin{array}{cc} d_{c,i_{ds}} & =\frac{\left(2i_{ds}-1\right)\cdot d_{r\max,i}}{2n_{di}}. \end{array}$$

Subsequently, assign *N* sampling points to $n_{di}$ distance subintervals and the number of the sampling points in the $i_{ds}$-th subintervals is $n_{di,i_{ds},total}$. For the $i_{ds}$-th subinterval, it is assumed that there are $n_{di,i_{ds},nlos}$ sampling points in the NLOS propagation. As such, the probability of NLOS propagation in the $i_{ds}$-th interval can be expressed as

(9) $$p_{di,i_{ds},nlos}=\frac{n_{di,i_{ds},nlos}}{n_{di,i_{ds},total}}.$$

The probability acquisition method related to distance is summarized in Algorithm 1.
**Algorithm 1** The probability acquisition method related to distance.**for**i←1 to *N* **do**    **for** $i_{ds}\leftarrow 1$ to $n_{di}$ **do**        $n_{di,i_{ds},total}\leftarrow 0$;        $n_{di,i_{ds},nlos}\leftarrow 0$;        **for**  j←1 to *N*  **do**           **if** $\left(i_{ds}-1\right)\cdot \frac{d_{r\max,i}}{n_{di}}<d_{r,i,j}\leq i_{ds}\cdot \frac{d_{r\max,i}}{n_{di}}$ **then**               $n_{di,i_{ds},total}\leftarrow n_{di,i_{ds},total}+1$;               **if** $g_{i,j}=1$ **then**                   $n_{di,i_{ds},nlos}\leftarrow n_{di,i_{ds},nlos}+1$;               **end if**           **end if**        **end for**        Calculate (9);    **end for****end for**

Similarly, the $i_{\theta s}$-th subinterval is $\left(-\pi +\left(i_{\theta s}-1\right)\cdot \frac{2\pi }{n_{\theta i}},-\pi +i_{\theta s}\cdot \frac{2\pi }{n_{\theta i}}\right]$ and the central point of the $i_{\theta s}$-th angle subinterval is described as

(10) $$\begin{array}{c} \theta_{c,i_{\theta s}}=-\pi +\frac{\pi \cdot (2i_{\theta s}-1)}{n_{\theta i}}. \end{array}$$

Then, assign *N* sampling points to $n_{\theta i}$ angle subintervals and the number of the sampling points in the $i_{\theta s}$-th subinterval is $n_{\theta i,i_{\theta s},total}$. Also, for the $i_{\theta s}$-th subinterval, it is assumed that there are $n_{\theta i,i_{\theta s},nlos}$ sampling points in the NLOS propagation. Consequently, the probability of NLOS propagation in the $i_{\theta s}$-th interval is calculated as

(11) $$p_{\theta i,i_{\theta s},nlos}=\frac{n_{\theta i,i_{\theta s},nlos}}{n_{\theta i,i_{\theta s},total}}.$$

The probability acquisition method related to angle is summarized in Algorithm 2.
**Algorithm 2** The probability acquisition method related to angle.**for**i←1 to *N* **do**    **for** $i_{\theta s}\leftarrow 1$ to $n_{\theta i}$ **do**        $n_{\theta i,i_{\theta s},total}\leftarrow 0$;        $n_{\theta i,i_{\theta s},nlos}\leftarrow 0$;        **for**  j←1 to *N*  **do**           **if** $-\pi +\left(i_{\theta s}-1\right)\cdot \frac{2\pi }{n_{\theta i}}<d_{r,i,j}\leq -\pi +i_{\theta s}\cdot \frac{2\pi }{n_{\theta i}}$ **then**               $n_{di,i_{\theta s},total}\leftarrow n_{\theta i,i_{\theta s},total}+1$;               **if** $g_{i,j}=1$ **then**                   $n_{\theta i,i_{\theta s},nlos}\leftarrow n_{\theta i,i_{\theta s},nlos}+1$;               **end if**           **end if**        **end for**        Calculate (11);    **end for****end for**

Based on the center points $d_{c,i_{ds}}$, $\theta_{c,i_{\theta s}}$ and the probabilities $p_{di,i_{s},nlos}$, $p_{\theta i,i_{s},nlos}$ in the NLOS propagation in each distance interval and angle interval, the polynomial curve fitting technique is used. Distance-NLOS probability polynomials and angle-NLOS probability polynomials are calculated as

(12) $$p_{di,nlos}=\sum_{{\bar{m}}_{i}=0}^{N_{{\bar{m}}_{i}}}a_{{\bar{m}}_{i}}\cdot d_{ai}^{{\bar{m}}_{i}},$$

(13) $$p_{\theta i,nlos}=\sum_{{\bar{n}}_{i}=0}^{N_{{\bar{n}}_{i}}}{\bar{a}}_{{\bar{n}}_{i}}\cdot \theta_{bi}^{{\bar{n}}_{i}},$$

where $p_{di,nlos}$ and $p_{\theta i,nlos}$ are the probabilities of the NLOS propagation at the distance $d_{ai}$ and the angle $\theta_{bi}$, respectively. $a_{{\bar{m}}_{i}}$ and ${\bar{a}}_{{\bar{n}}_{i}}$ are the fitted coefficients, which can be achieved by least square polynomial fitting method. It can be expressed as

(14) $$\begin{array}{c} min\sum_{i_{ds}=1}^{n_{di}}{\left[p_{di,nlos}\left(d_{c,i_{ds}}\right)-p_{di,i_{ds},nlos}\right]}^{2}, \end{array}$$

where $p_{di,nlos}\left(d_{c,i_{ds}}\right)=\sum_{{\bar{m}}_{i}=0}^{N_{{\bar{m}}_{i}}}a_{{\bar{m}}_{i}}\cdot d_{c,i_{ds}}^{{\bar{m}}_{i}}$. By taking partial derivatives of (14) with respect to $a_{{\bar{m}}_{i}}$ and setting them equal to 0, we have

(15) $$\begin{array}{c} \left\{\begin{array}{ccc} \; & 2\sum_{i_{ds}=1}^{n_{di}}\left[p_{di,nlos}\left(d_{c,i_{ds}}\right)-p_{di,i_{ds},nlos}\right] & =0\\ \; & 2\sum_{i_{ds}=1}^{n_{di}}\left[p_{di,nlos}\left(d_{c,i_{ds}}\right)-p_{di,i_{ds},nlos}\right]d_{c,i_{ds}} & =0\\ \; & \;\;\;\;\;\;\;\;\;\;\;\;\;\;\;\;\;\;\;\;\vdots \\ \; & 2\sum_{i_{ds}=1}^{n_{di}}\left[p_{di,nlos}\left(d_{c,i_{ds}}\right)-p_{di,i_{ds},nlos}\right]d_{c,i_{ds}}^{N_{{\bar{m}}_{i}}} & =0 \end{array}\right.\;\;\;. \end{array}$$

Equation (15) can be organized into a matrix form as follows:

(16) $$\begin{array}{c} \left[\begin{array}{cccc} n_{di} & \sum_{i_{ds}=1}^{n_{di}}d_{c,i_{ds}} & \cdots & \sum_{i_{ds}=1}^{n_{di}}d_{c,i_{ds}}^{N_{{\bar{m}}_{i}}}\\ \sum_{i_{ds}=1}^{n_{di}}d_{c,i_{ds}} & \sum_{i_{ds}=1}^{n_{di}}d_{c,i_{ds}}^{2} & \cdots & \sum_{i_{ds}=1}^{n_{di}}d_{c,i_{ds}}^{N_{{\bar{m}}_{i}}+1}\\ \vdots & \vdots & \ddots & \vdots \\ \sum_{i_{ds}=1}^{n_{di}}d_{c,i_{ds}}^{N_{{\bar{m}}_{i}}} & \sum_{i_{ds}=1}^{n_{di}}d_{c,i_{ds}}^{N_{{\bar{m}}_{i}}+1} & \cdots & \sum_{i_{ds}=1}^{n_{di}}d_{c,i_{ds}}^{2N_{{\bar{m}}_{i}}} \end{array}\right]\left[\begin{array}{c} a_{0}\\ a_{1}\\ \vdots \\ a_{N_{{\bar{m}}_{i}}} \end{array}\right]=\left[\begin{array}{c} \sum_{i_{ds}=1}^{n_{di}}p_{di,i_{ds},nlos}\\ \sum_{i_{ds}=1}^{n_{di}}d_{c,i_{ds}}p_{di,i_{ds},nlos}\\ \vdots \\ \sum_{i_{ds}=1}^{n_{di}}d_{c,i_{ds}}^{N_{{\bar{m}}_{i}}}p_{di,i_{ds},nlos} \end{array}\right]. \end{array}$$

It can be expressed as

(17) $$\begin{array}{c} F^{T}Fa=F^{T}y, \end{array}$$

where $F=\left[\begin{array}{cccc} 1 & d_{c,1} & \cdots & d_{c,1}^{N_{{\bar{m}}_{i}}}\\ 1 & d_{c,2} & \cdots & d_{c,2}^{N_{{\bar{m}}_{i}}}\\ \vdots & \vdots & \cdots & \vdots \\ 1 & d_{c,n_{di}} & \cdots & d_{c,n_{di}}^{N_{{\bar{m}}_{i}}} \end{array}\right]$, $y=\left[\begin{array}{c} p_{di,1,nlos}\\ p_{di,2,nlos}\\ \vdots \\ p_{di,n_{di},nlos} \end{array}\right]$ and $a=\left[\begin{array}{c} a_{0}\\ a_{1}\\ \vdots \\ a_{N_{{\bar{m}}_{i}}} \end{array}\right]$.

Therefore, Equation (17) can be solved as

(18) $$\begin{array}{c} a={\left(F^{T}F\right)}^{-1}F^{T}y. \end{array}$$

Similarly, the coefficients ${\bar{a}}_{{\bar{n}}_{i}}$ can be achieved as

(19) $$\begin{array}{c} \bar{a}={\left(G^{T}G\right)}^{-1}G^{T}\bar{y}, \end{array}$$

where $G=\left[\begin{array}{cccc} 1 & \theta_{c,1} & \cdots & \theta_{c,1}^{N_{{\bar{n}}_{i}}}\\ 1 & \theta_{c,2} & \cdots & \theta_{c,2}^{N_{{\bar{n}}_{i}}}\\ \vdots & \vdots & \cdots & \vdots \\ 1 & \theta_{c,n_{\theta i}} & \cdots & \theta_{c,n_{\theta i}}^{N_{{\bar{n}}_{i}}} \end{array}\right]$, $\bar{y}=\left[\begin{array}{c} p_{\theta i,1,nlos}\\ p_{\theta i,2,nlos}\\ \vdots \\ p_{\theta i,n_{\theta i},nlos} \end{array}\right]$, and $\bar{a}=\left[\begin{array}{c} {\bar{a}}_{0}\\ {\bar{a}}_{1}\\ \vdots \\ {\bar{a}}_{N_{{\bar{n}}_{i}}} \end{array}\right]$.

The probability of the NLOS propagation between the point (x,y) and the *i*-th anchor is proposed as:

(20) $$\begin{array}{cc} p_{i,nlos} & =\alpha_{i}\cdot p_{di,nlos}+\left(1-\alpha_{i}\right)\cdot p_{\theta i,nlos}, \end{array}$$

where

(21) $$\begin{array}{cc} \alpha_{i} & =\frac{1}{2}\left(e^{-k_{0}\cdot d_{i}}+1\right), \end{array}$$

(22) $$\begin{array}{cc} d_{i} & =\sqrt{{\left(x_{i}-x\right)}^{2}+{\left(y_{i}-y\right)}^{2}}, \end{array}$$

(23) $$\begin{array}{cc} \theta_{i} & =a\tan2(y_{i}-y,x_{i}-x) \end{array}$$

and $k_{0}$ is a positive constant.

**Remark** **2.** *In this paper, a new probability model (21) is put forward which is more reasonable to reflect the practical engineering. It is not difficult to see that when the difference of the distance between the estimated position and the real position is fixed, angle error increases as the distance between the real position and the position of the anchor decreases. Therefore, the smaller the distance is, the smaller the confidence level of the angle-based estimation is. In addition, the exponential form of parameter $\alpha_{i}$ is selected by experiments.*

## 4. Simplified Calculation of Maximum Likelihood Estimation

According to (4), the probability density function of the distance between each anchor and the target can be obtained. Assuming that the probability density function of the distance between each anchor and the target is independent, one has the following joint likelihood function:

(24) $$p\left(z;x\right)=\prod_{i=1}^{n}p(z_{i};x).$$

The estimate of *x* by MLE can be obtained as:

(25) $$\begin{array}{cc} {\hat{X}}_{MLE} & =\arg\max_{x}\ln p\left(z;x\right)\\ \; & =\arg\max_{x}\sum_{i=1}^{n}\ln p(z_{i};x), \end{array}$$

where ${\hat{X}}_{MLE}={\left[{\hat{x}}_{MLE},{\hat{y}}_{MLE}\right]}^{T}$.

Normally, finding the analytical solution of (25) is quite time-consuming, which deteriorates the real-time performance of the system to a great extent. For the purpose of overcoming such a difficulty, an improved Monte Carlo method is proposed to solve (25), which could reduce the computational complexity and accordingly improve the real-time performance of the system.

Assume that at the time k−1, the position and velocity of the target are $\left(x_{k-1},y_{k-1}\right)$ and $\left(v_{x,k-1},v_{y,k-1}\right)$, respectively. The angle between the velocity direction and the positive direction of the *x*-axis is calculated as

(26) $$\theta_{v,k-1}=a\tan2\left(v_{y,k-1},v_{x,k-1}\right),$$

where $a\tan2(v_{y,k-1},v_{x,k-1})$ is the angle between the vector from origin to $\left(v_{x,k-1},v_{y,k-1}\right)$ and *x*-axis positive direction vector. For the convenience of discussion, the position of the point is expressed in polar coordinates, and $\left(x_{k-1},y_{k-1}\right)$ is used as the coordinate origin of polar coordinates. Therefore, the points can be obtained from the parameters of $d_{m,i}$ and $\theta_{m,i}$ in terms of the following two methods, respectively.

(a) The distance satisfies $d_{m,i}∼N\left(0,\sigma_{{}_{ma}}^{2}\right)$ and the angle satisfies $\theta_{m,i}∼U\left(-\pi ,\pi \right)$;

(b) The angle satisfies $\theta_{m,i}∼N\left(\theta_{v,k-1},\sigma_{{}_{mb}}^{2}\right)$ and the distance satisfies $d_{m,i}∼U\left(0,d_{m,\max}\right)$, where $\sigma_{{}_{ma}}^{2}$ and $\sigma_{{}_{mb}}^{2}$ are the variance of distance and angle, respectively, $d_{m,\max}$ represents the upper bound of distance.

The point of maximum value solved in (24) is taken as the optimal estimation point.

## 5. Localization Based on Extended Kalman Filter Algorithm

The motion model is represented as a discrete-time system and decomposed into state vectors in both directions *x* and *y*, which can be expressed as

(27) $$\left[\begin{array}{c} x(k)\\ y(k)\\ v_{x}\left(k\right)\\ v_{y}\left(k\right) \end{array}\right]=\left[\begin{array}{cccc} 1 & 0 & \Delta t & 0\\ 0 & 1 & 0 & \Delta t\\ 0 & 0 & 1 & 0\\ 0 & 0 & 0 & 1 \end{array}\right]\left[\begin{array}{c} x(k-1)\\ y(k-1)\\ v_{x}\left(k-1\right)\\ v_{y}\left(k-1\right) \end{array}\right],$$

where x(k), $v_{x}\left(k\right)$ are the position and velocity of target in the *x*-axis and y(k), $v_{y}\left(k\right)$ are the position and velocity of target in the *y*-axis of the system at time *k*.

Therefore, the prediction phase of the Kalman filter can be expressed as

(28) $$\begin{array}{cc} X_{k|k-1} & =AX_{k-1|k-1}, \end{array}$$

(29) $$\begin{array}{cc} P_{k|k-1} & =AP_{k-1|k-1}A^{T}+Q, \end{array}$$

where $A=\left[\begin{array}{cccc} 1 & 0 & \Delta t & 0\\ 0 & 1 & 0 & \Delta t\\ 0 & 0 & 1 & 0\\ 0 & 0 & 0 & 1 \end{array}\right]$.

$X_{k-1|k-1}={\left[x\left(k-1\right),y\left(k-1\right),v_{x}\left(k-1\right),v_{y}\left(k-1\right)\right]}^{T}$, $X_{k-1|k-1}$ is optimal estimate of state vector *X* at time k−1, $P_{k-1|k-1}$ is the posteriori error covariance matrix at time k−1. *Q* is the covariance matrix of the process noise which is set to a diagonal matrix.

The prediction distance vector between anchors and the target is derived as

(30) $$h\left(X_{k|k-1}\right)=\left[\begin{array}{c} \sqrt{{\left(x_{k|k-1}-x_{1}\right)}^{2}+{\left(y_{k|k-1}-y_{1}\right)}^{2}}\\ \vdots \\ \sqrt{{\left(x_{k|k-1}-x_{i}\right)}^{2}+{\left(y_{k|k-1}-y_{i}\right)}^{2}}\\ \vdots \\ \sqrt{{\left(x_{k|k-1}-x_{n}\right)}^{2}+{\left(y_{k|k-1}-y_{n}\right)}^{2}} \end{array}\right],$$

where $\left(x_{k|k-1},y_{k|k-1}\right)$ is the position estimation of the target in the prediction phase. Moreover, the Jacobian matrix of (30) is obtained as

(31) $$H_{k}=\left[\begin{array}{cccc} \frac{x_{k|k-1}-x_{1}}{h_{1}\left(X\right)} & \frac{y_{k|k-1}-y_{1}}{h_{1}\left(X\right)} & 0 & 0\\ \vdots & \vdots & \vdots & \vdots \\ \frac{x_{k|k-1}-x_{i}}{h_{i}\left(X\right)} & \frac{y_{k|k-1}-y_{i}}{h_{i}\left(X\right)} & 0 & 0\\ \vdots & \vdots & \vdots & \vdots \\ \frac{x_{k|k-1}-x_{n}}{h_{n}\left(X\right)} & \frac{x_{k|k-1}-x_{n}}{h_{n}\left(X\right)} & 0 & 0 \end{array}\right].$$

The filter gain matrix $K_{k}$ is determined by

(32) $$K_{k}=P_{k|k-1}{H_{k}}^{T}\left(H_{k}P_{k|k-1}{H_{k}}^{T}+R\right),$$

where *R* represents the covariance matrix of the observation noise which is set to a diagonal matrix.

The update phase of the Kalman filter is given as follows:

(33) $$X_{k|k}=X_{k|k-1}+K_{k}\left({d_{k}}^{\prime }-h_{k}\right),$$

(34) $$P_{k|k}=P_{k|k-1}-K_{k}H_{k}P_{k|k-1},$$

where $P_{k|k}$ is the posteriori error covariance matrix at time *k*, $X_{k|k}$ represents the posteriori state estimate at time *k*, and ${d_{k}}^{\prime }$ represents corrected measuring distance vector from the MLE method at time *k* with the following form:

(35) $${d_{k}}^{\prime }=\left[\begin{array}{c} \sqrt{{\left({\hat{x}}_{MLE}-x_{1}\right)}^{2}+{\left({\hat{y}}_{MLE}-y_{1}\right)}^{2}}\\ \vdots \\ \sqrt{{\left({\hat{x}}_{MLE}-x_{i}\right)}^{2}+{\left({\hat{y}}_{MLE}-y_{i}\right)}^{2}}\\ \vdots \\ \sqrt{{\left({\hat{x}}_{MLE}-x_{n}\right)}^{2}+{\left({\hat{y}}_{MLE}-y_{n}\right)}^{2}} \end{array}\right].$$

## 6. Simulation and Experimental Results

### 6.1. Simulation Results

UWB technology is widely used in indoor localization because it is insensitive to channel fading and has high positioning accuracy. Therefore, we simulated UWB communication to verify the effectiveness of the algorithm. In this section, an indoor environment with obstacles is considered, where the test area was a rectangular space of 14 m × 5 m. There were eight anchors assigned around the space, whose locations were respectively known as [0 cm, 0 cm], [500 cm, −100 cm], [1000 cm, 0 cm], [1100 cm, 150 cm], [1000 cm, 300 cm], [500 cm, 400 cm], [0 cm, 300 cm], [−100 cm, 150 cm]. The obstacle was considered in the space and the location of the obstacle was unknown and fixed. It was assumed that NLOS error occurred when the connecting line of the target to the anchor traverses the obstacle, otherwise there was no NLOS error. The LOS error obeyed $\varepsilon_{L}∼N\left(0\;\mathrm{cm},2.6\;\mathrm{cm}\right)$ and NLOS error obeyed $\varepsilon_{NL}∼N\left(22\;\mathrm{cm},3.6\;\mathrm{cm}\right)$. In the simulation, the proposed algorithm was compared with the existing ones; see Table 1.

**Figure 1:**
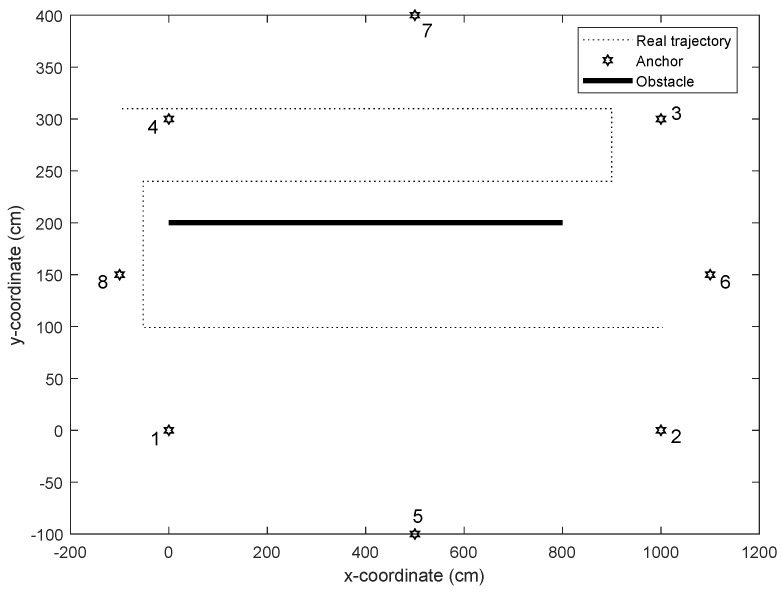
Simulation environment setting and real trajectory.

Figure 1 shows the simulation environment setting and real trajectory. The obstacle was placed in the middle of the environment and anchors were placed around. In the test, the target moved along a black dotted line, which was S-shaped. The velocity of the target was set to 0.3 m/s ∼0.5 m/s. In order to test the performance of algorithms in Table 1, the root mean square error (RMSE) was used as the main performance metric. It was defined as $RMSE=\sqrt{(\sum_{i=1}^{T_{n}}\left|\right|x_{r,i}-{\hat{x}}_{i}{\left|\right|}^{2})/T_{n}}$, where $x_{r,i}$ denotes the true position of the target at time *i*, ${\hat{x}}_{i}$ indicates the estimated position.

**Figure 2:**
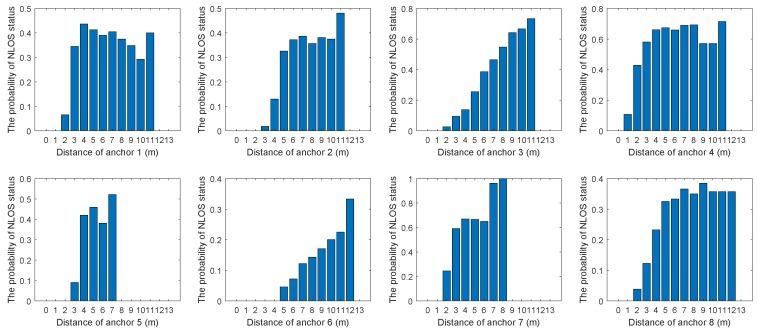
The relationship between the probability of non-line-of-sight (NLOS) status and distance in eight anchors.

**Figure 3:**
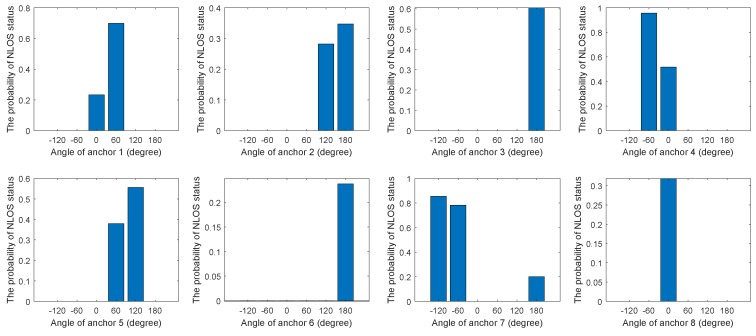
The relationship between the probability of NLOS status and angle in eight anchors.

Before using the proposed algorithm, it was necessary to estimate the probability of occurrence of the NLOS status at different distances and angles by sampling. Figure 2 and Figure 3 show the relationship between the probability of NLOS status and distance or angle in eight anchors. As can be seen from Figure 2, in general, the larger the distance, the higher the probability of NLOS status. From Figure 3, the relationship between the angle and the probability of NLOS status could be obtained. It was obviously different in different environments. According to Figure 2 and Figure 3, polynomial fitting could be used to obtain the relationship between NLOS status occurrence probability and distance or angle. Alternatively, the relationship could be obtained by constructing a piecewise function.

**Figure 4:**
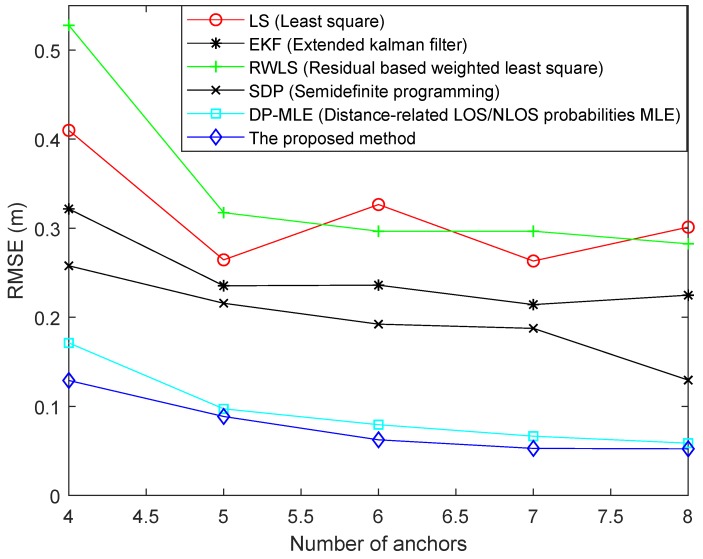
Root mean square error (RMSE) of different algorithms using different numbers of anchors.

In order to reduce the operation time, 500 points were randomly selected according to the introduced Monte Carlo method and one point was selected which could maximize Formula (24). Figure 4 illustrates the RMSE of different algorithms with different numbers of anchors. The distribution of noise was the same in each algorithm experiment. As can be seen from Figure 4, as the number of anchors increased, the RMSE of algorithms gradually decreased, except for the LS algorithm. When the number of anchors was 6 or 8, the RMSE of the LS algorithm was increased. The reason may be that NLOS measurement error of the added anchor was large, which led to a large error. Since the weights of LS algorithm were equal, the algorithm could not allocate less weight to the measurement with larger NLOS error. On the contrary, the RWLS algorithm compensated for this deficiency. The algorithm assigned weights to the measurements of each anchor by analyzing the positioning residuals of each anchor. However, the algorithm could not exert its advantages when the number of anchors was small. Therefore, when the number of anchors was four or five, the RMSE was higher than that of the LS method. The RMSE of the EKF algorithm was the highest when the number of anchors was four. As this number increased, the RMSE gradually stabilized, but the value was always higher than some other methods, because it does not suppress NLOS error very well. The SDP method worked well when the number of NLOS status was large or small in all measurements. From the figure, it worked better than the EKF method. The DP-MLE method uses statistical methods to estimate the relationship between the probability of NLOS propagation and the measured distance. However, the proposed method not only considers the relationship between its probability of occurrence and the measured distance, but also considers the relationship with measurement angle. Therefore, the RMSE of the proposed algorithm was smaller than that of the DP-MLE method.

**Figure 5:**
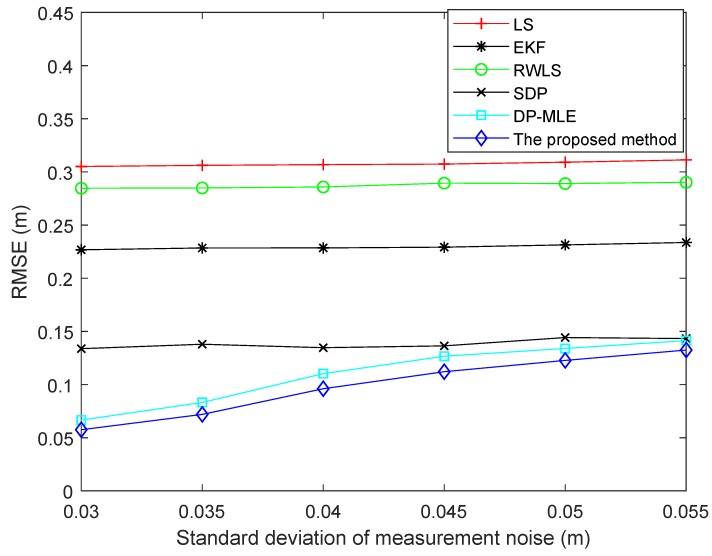
RMSE of different algorithms using different standard deviations of measurement noise.

Figure 5 shows the RMSEs of different algorithms using different standard deviations of measurement noise. It can be seen from the figure that the RMSE of the proposed method was the lowest and that of the LS method was the highest in different standard deviations. Since the RWLS algorithm assigns less weight to anchors with large residuals, the RMSE of the method was lower than that of the LS method. The advantage of the EKF method is mainly its ability to suppress the measurement noise; consequently, the RMSE of the method was lower than that of RWLS. The SDP algorithm estimates the average value of NLOS error and constructs the model based on statistical features. As a result, the method was superior to the EKF method. The RMSE of the proposed method and DP-MLE method both increased with increasing standard deviation. This is why the constructed models are probability models based on NLOS status. When the standard deviation of measurement noise increased, the accuracy of NLOS status judgment will be reduced. For example, when the measurement noise is larger than NLOS error, it may be considered as NLOS noise. However, the RMSE of the proposed algorithm was still lower than those of DP-MLE and SDP methods, based on Figure 5.

**Figure 6:**
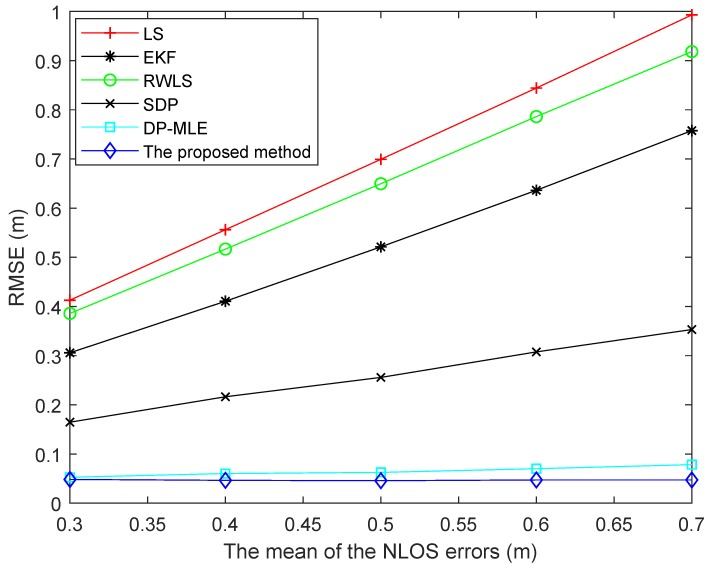
RMSEs of different algorithms using different means of NLOS error.

Figure 6 shows the RMSEs of different algorithms using different means of NLOS error. With the increase of the mean of NLOS error, the RMSEs of LS, EKF, and RWLS algorithms increased obviously and gradually. The main reason is that these methods could not effectively suppress the NLOS error. The SDP method can suppress the NLOS error without identifying NLOS status. Both DP-MLE and the proposed method could estimate the probability of NLOS status. However, the accuracy of the proposed method was higher than that of DP-MLE method. Therefore, the RMSE of this method was lower than that of DP-MLE method.

### 6.2. Experimental Results

In order to better verify the effectiveness of the algorithm, we used the measurement database in [31]. The experimental equipment consisted of 20 anchor nodes and one mobile node, which were UWB devices. Some anchor nodes were placed in the corridor and the other parts were placed in the room. The two-way arrival time estimation method was used in ranging. The mobile node was placed on the mobile robot, which was placed on the orbit. Due to the influence of energy attenuation in propagation, the mobile node could not accept all signals of anchor nodes. Therefore, we selected nine anchor nodes that could successfully communicate with the mobile node for the experiment; see Table 2.

In the experiment, the height of the target was fixed and known, and the height of the mobile node was set to 0.162 m. Since the algorithm was calculated in a two-dimensional environment, the measured data needed to be converted. According to (36), the measured distance was converted into the horizontal distance between the target and the anchor.

(36) $$d=\sqrt{d_{m}^{2}-\Delta h^{2}},$$

where $d_{m}$ is the measured distance and Δh is the height difference between the mobile node and the anchor node.

**Figure 7:**
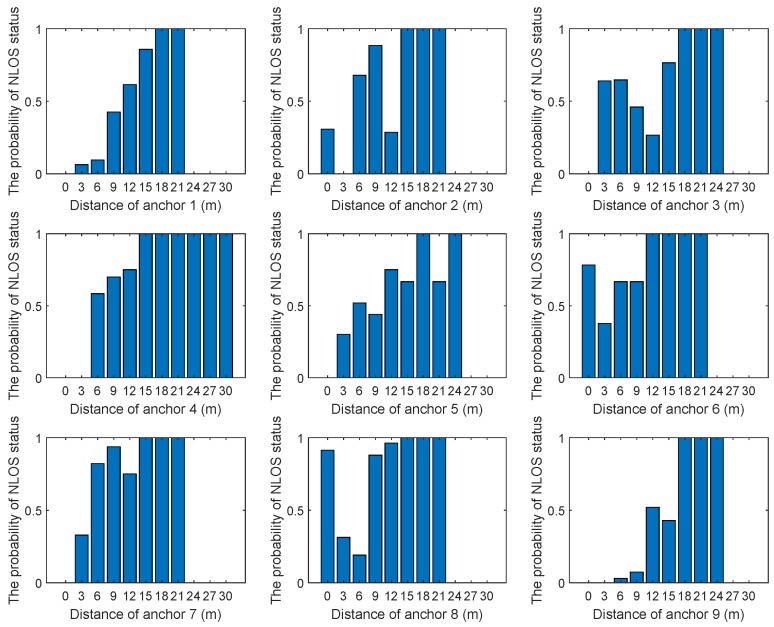
The relationship between distance and NLOS status probability. The horizontal axis of each graph represents distance (m) and the vertical axes represent NLOS status probability.

**Figure 8:**
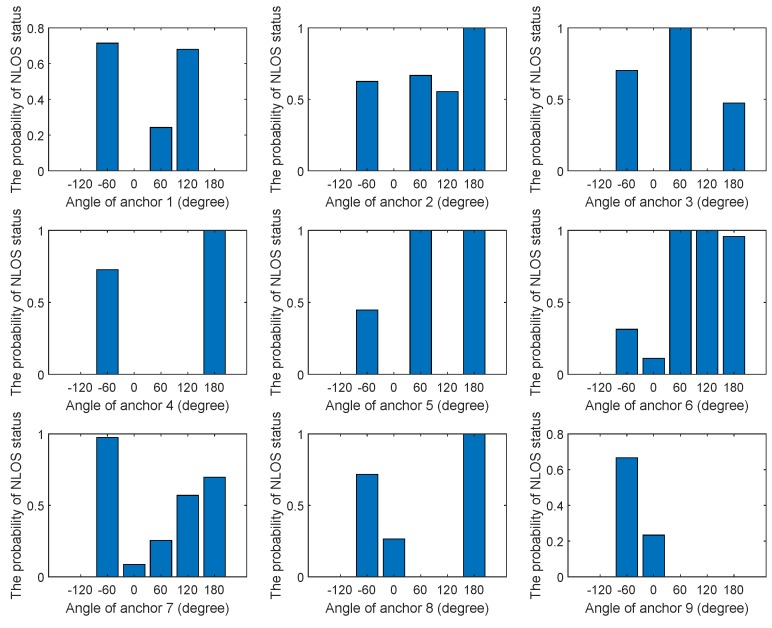
The relationship between angle and NLOS status probability. The horizontal axis of each graph represents angle (degree) and the vertical axes represent NLOS status probability.

The parameters of NLOS propagation occurrence probability model need to be obtained by sampling before testing; that is, $p_{los}\left(z_{i}\right)$ and $p_{nlos}\left(z_{i}\right)$. In the data set, about 90% of the data was used for training and about 10% for testing. The distances between each anchor and the remaining 19 anchors were measured. However, the communication between some anchors could not be established. Therefore, the measurements of two anchors that could not establish communication were set as NLOS propagation. Since the positions of all anchors were known, the distance between any two anchors could be calculated. In order to reduce the measurement errors caused by faults or other factors, multiple measurements were used and the average value was calculated as the final measurement value. If the difference between the measured value and the true distance exceeded the threshold, the measurement was considered as the NLOS propagation. The relationship between distance, the angle of each sampling point, and the NLOS status probability is given in Figure 7 and Figure 8.

**Figure 9:**
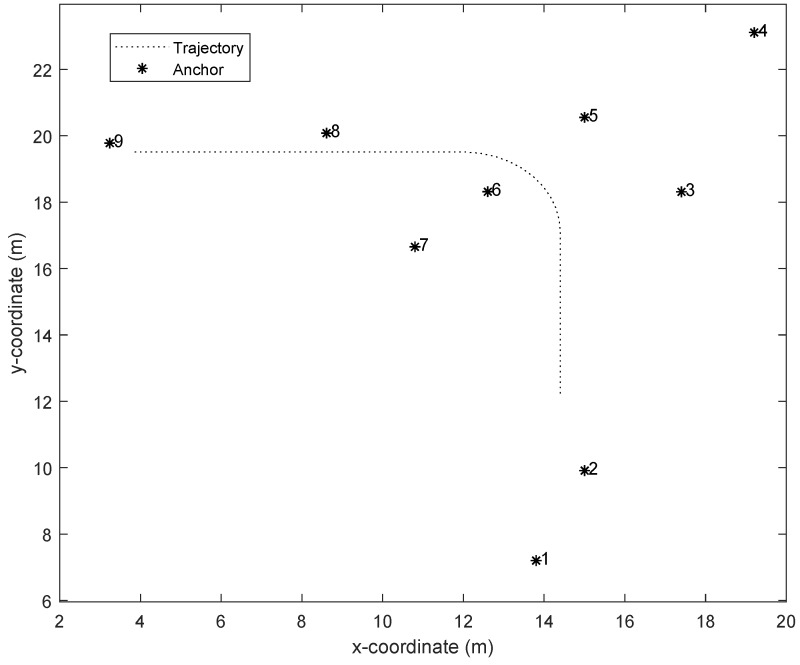
The robot trajectory and the position of anchors.

**Figure 10:**
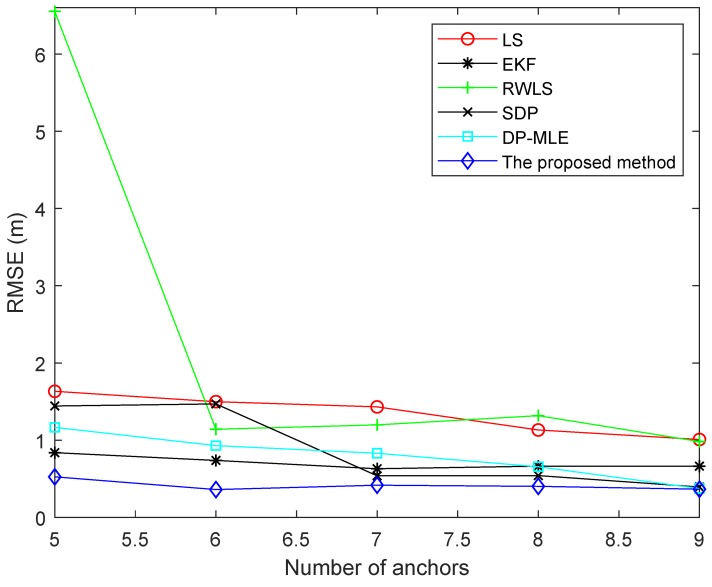
RMSEs of different algorithms using different numbers of anchors.

In the test, we used the data of 20 s of target movement to complete the experiment. The robot trajectory and the position of anchors is given in Figure 9. The mobile robot moved at a constant speed and turned left at about time 8 s. The RMSEs of different numbers of anchors are given in Figure 10.

As can be seen from Figure 10, the RWLS method had the worst effect when the number of anchors was five. Because the RWLS method could not accurately identify NLOS error when the number of anchors was small, it led to the wrong weight. The performance of the proposed method and the EKF method did not change much as the number of anchors increased, but the performance of the proposed method was better than that of other methods. The reason is that the proposed method could effectively suppress the NLOS errors when the number of anchors was small. When the number of anchors was 6 or 7, the weight of the RWLS method was accurate. Therefore, its effect was better than that of the LS method. However, the performance of these two methods was inferior to the EKF method and the proposed method because the two methods do not consider suppressing measurement errors. When the number of anchors was nine, the DP-MLE, SDP, and proposed method could effectively suppress NLOS error, but the DP-MLE and SDP methods found it difficult to identify NLOS errors when the number of anchors was small. In addition, the SDP algorithm is solved by interior point method, and its operation time was much longer than that of the proposed algorithm. As a result, the real-time performance of the SDP algorithm Was far inferior to that of the proposed algorithm.

## 7. Conclusions

In the paper, an optimization algorithm is presented based on a distance and angle probability model for indoor non-line-of-sight (NLOS) environments. According to the relationship between distance, angle, and the occurrence probability of NLOS propagation, the joint likelihood function was established. The simulation and experimental results showed that the NLOS error was compensated effectively in NLOS propagation, and the proposed algorithm was superior to other algorithms when the number of anchors was small. Furthermore, the real-time performance of the proposed algorithm was much higher than that of SDP algorithm and the accuracy of the algorithm was ensured.

## Figures and Tables

**Table 1:** The list of the considered algorithms and descriptions.

Algorithm	Description
LS	Least square method
EKF	Extended kalman filtering algorithm [29]
RWLS	Residual based weighted least square algorithm [30]
SDP	Semidefinite programming method [16]
DP-MLE	Distance-related LOS/NLOS probabilities maximum likelihood estimation [14]
DAP-MLE	The proposed method

**Table 2:** The list of the locations of anchor nodes.

Anchor ID	Coordinate X (m)	Coordinate Y (m)	Coordinate Z (m)
8	13.80	7.20	1.13
9	15.00	9.91	1.13
11	17.40	18.31	1.13
12	19.21	23.11	1.13
13	15.00	20.56	1.13
14	12.60	18.31	1.13
15	10.80	16.65	1.13
16	8.61	20.08	1.13
17	3.24	19.78	1.13
